# The Reward of the Philanthropic Physician

**Published:** 1900-08

**Authors:** 


### Abstracts and Selections.

### The Reward of the Philanthropic Physician.

*Wilkinson's Medical Digest* cites the following from the *Boston Medical and Surgical Journal* of unspecified date: "A physician in this vicinity was recently called to a family which he found in such destitute circumstances that he gave, in addition to his prescription, a five-dollar bill. Happening in the next day, he discovered that his gift had been thus spent: "Three dollars to the priest, and two dollars to get another doctor.—*N. Y. Medical Journal.*

